# Prenatally detected six duplications at Xp22.33-p11.22: a case report

**DOI:** 10.1186/s12884-023-05627-0

**Published:** 2023-04-27

**Authors:** Xue Zhang, Jian Li, Lan Zhang, Hongli Liu, Hong Yi, Mingxing Liang, Jianyu Luo, Junnan Li, Yanling Dong

**Affiliations:** 1 Department of Obstetrics, The First Affiliated Hospital of Chongqing Medical University, 1 Youyi Road, Yuzhong, Chongqing, 400016 People’s Republic of China; 2 Chongqing Fetal Medical Centre, The First Affiliated Hospital of Chongqing Medical University, Chongqing, 400016 People’s Republic of China

**Keywords:** Congenital heart defects, Copy number variations, Chromosome microarray analysis, Multiple ligation-dependent probe amplification, Single nucleotide polymorphisms

## Abstract

**Background:**

The discrepancy between the results of cytogenetics and the results of chromosome microarray analysis (CMA) has often led to confusion over genetic counselling for prenatal diagnosis.

**Case presentation:**

The prenatal ultrasound results of a congenital heart defect (CHD) foetus displayed an apartial endocardial pad defect and permanently dilated coronary sinus and left superior vena cava at 21 weeks of gestation. Cytogenetic analysis, CMA, fluorescent in situ hybridization (FISH) and multiplex ligation-dependent probe amplification (MLPA) with foetal cord blood samples were used to detect the genetic aetiology. Routine G-binding cytogenetic analysis showed normal karyotypes in both the foetus’ and parents’ blood samples. CMA results demonstrated that there were 53.973-Mb recurrent CNVs at Xp22.33-p11.22, as confirmed by MLPA assay.

**Conclusions:**

Herein, we described the CNV of six duplications at Xp22.33-p11.22 and the 53.973 Mb duplication CNV that was not found in foetal cord blood samples by conventional cytogenetic methods, and it was confirmed by CMA and MLPA. Our novel findings will provide helpful information for prenatal diagnosis and genetic counselling for foetal CHDs.

## Introduction

While standard cytogenetic evaluation revealed only normal karyotypes in the foetal cells investigated, the explanation of abnormal foetal phenotypes, except for environmental and chromosomal abnormalities, cannot exclude false-negatives due to the limitations of this technique. The discrepancy between the results of cytogenetics and the results of chromosome microarray analysis (CMA) has often led to confusion over genetic counselling for prenatal diagnosis.

CMA and Multiple ligation-dependent probe amplification (MLPA) without cell culture can uncover some cytogenetic abnormalities that cannot be found by standard cytogenetic valuation. In addition, congenital heart defects (CHDs) are structural anomalies of the heart or blood vessels that arise during cardiac embryogenesis. Several studies have indicated that CNVs are the major genetic cause of cardiovascular disease.

Here, we describe a case of six duplications at Xp22.33-p11.22 for foetal CHDs detected in cord blood cells but not confirmed by conventional chromosome analysis, which, in theory, duplication of such a 53.973 Mb fragment should be detectable at the chromosomal level. Our novel findings will provide useful information for prenatal diagnosis and genetic counselling for foetal CHDs.

## Case description

A 30-year-old woman, gravida 3 para 0, was included in this study. There was no prenatal diagnosis for two previous pregnancies, one ectopic pregnancy and one missed abortion. Neither the pregnant woman nor her husband had a family history of genetic diseases. Foetal CHDs were diagnosed by routine prenatal ultrasound at 21 weeks of gestation. Umbilical cord blood and parents’ peripheral blood were collected after written informed consent was obtained. G-banding karyotype analysis with peripheral blood samples were collected from the parents. Cytogenetic analysis was performed according to the standard protocol with a 400-band resolution.

In the CMA experiment, the detection of CNVs was applied with the use of Infinium Global Screening Array (Illumina, San Diego, CA) comprising 650 K Oligo Probes. Peripheral blood from parent and foetal cord blood samples was applied for the following measurement. A QIAamp DNA Mini Kit (Qiagen, Hilden, Germany) was used to extract the DNA. This microarray was constructed for the sole purpose of identifying DNA copy number gains and losses associated with chromosomal imbalances. The array was scanned by the Illumina iScan microarray scanning system. Raw data were uploaded in KaryoStudio 1.4.3.0 Build 37 software (Illumina, San Diego, CA), and log R ratios and BAFs were calculated by normalization to a reference ‘cluster’, which was generated from a set of 2000 ~ 3000 clinical samples.

The MLPA was applied by Chengdu Precision Medicine Laboratory following the manufacturer’s instructions [1]. DNA was isolated from foetal cord blood for further detection. MLPA (P160) was utilized to detect gene exon duplications in the sample to be tested, and normal DNA was used as a control. Twenty-three probes of *NLGN4X*, *PUDP*, *STS*, *ANOS1* and *GPR143* exon located at Xp22.32 in the kit were included for detection. Data were analysed using Coffalyser Software from MRC-Holland (Amsterdam, The Netherlands). The results were considered to have six duplications when the ratio was between 3.0 and 3.5. Fluorescent in situ hybridization (FISH) was performed using standard protocols with an Aneuploidy analysis kit (Abbott Molecular Inc., Des Plaines, IL, USA) with the cells from the nonculture cord blood samples located within the X/Y,18 chromosomal centromere region. Data analysis was performed using a BX61 Olympus epifluorescence microscope (Olympus, Tokyo, Japan) with Applied Spectral Imaging FISH View 6.0 software (Applied Spectral Imaging, Inc., Carlsbad, CA, USA). The chromosome region with the information was provided by the DECIPHER Database (http://decipher.sanger.ac.uk), the Online Mendelian Inheritance in Man database (http://omim.org/), ClinGen database (http://dosage.clinicalgenome.org/), DGV database (http://dgv.tcag.ca/dgv/app/home), the UCSC database (http://genome.ucsc.edu) and the National Venter for Biotechnology Information (https://pubmed.ncbi).

**Fig. 1:**
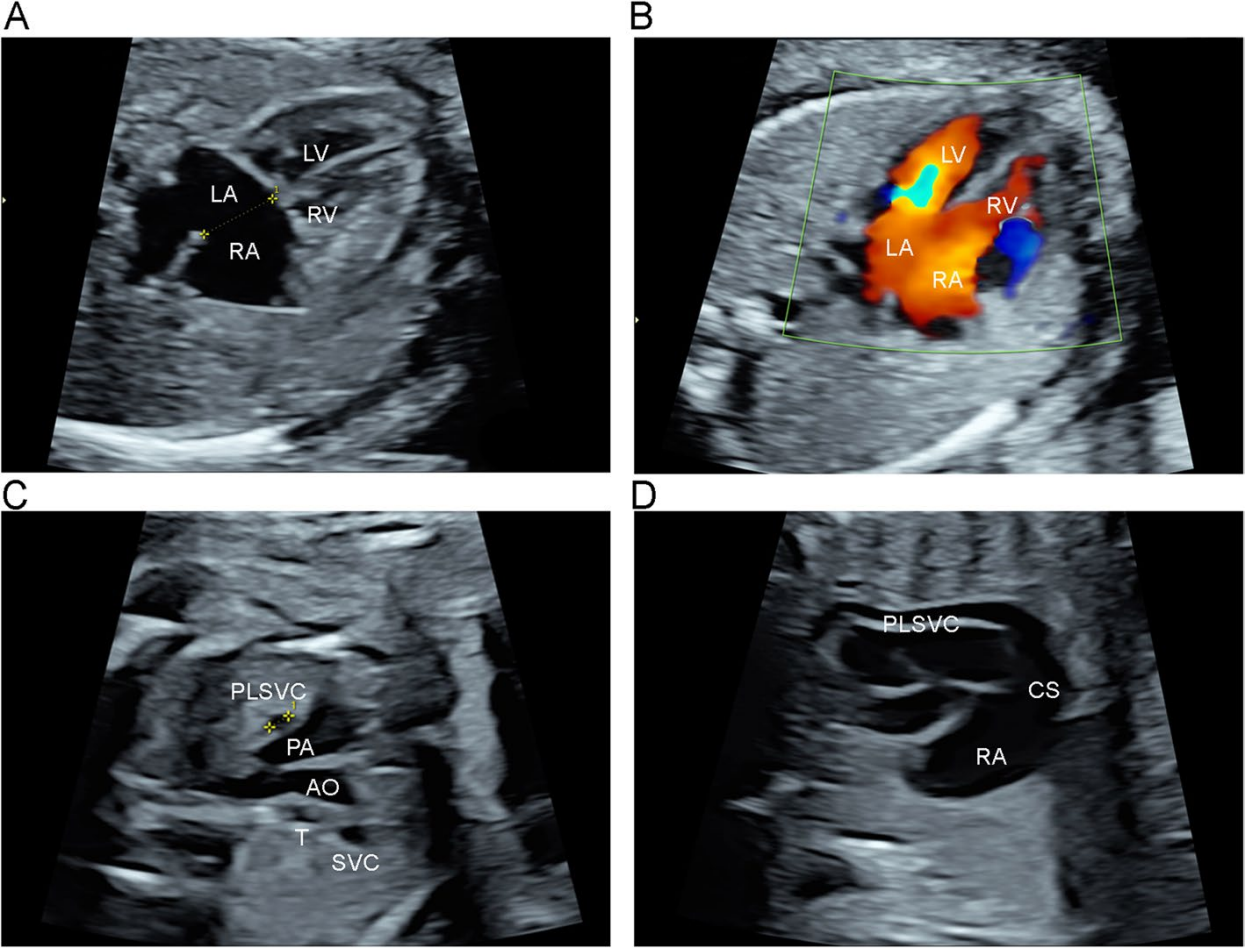
The results of prenatal ultrasound. **A** The primary atrial septal defect and (**B**) the communication of blood between the left and right atrium. **C** The vascular shadow on the left side of the pulmonary artery and (**D**) merge into the dilated coronary sinus. LA left atrium; RA, right atrium; LV, left ventricle; RV, right ventricle; PLSVC, perpetual left superior vena cava; PA, pulmonary artery; AO, aorta; T, trachea; SVC, superior vena cava

**Fig. 2:**
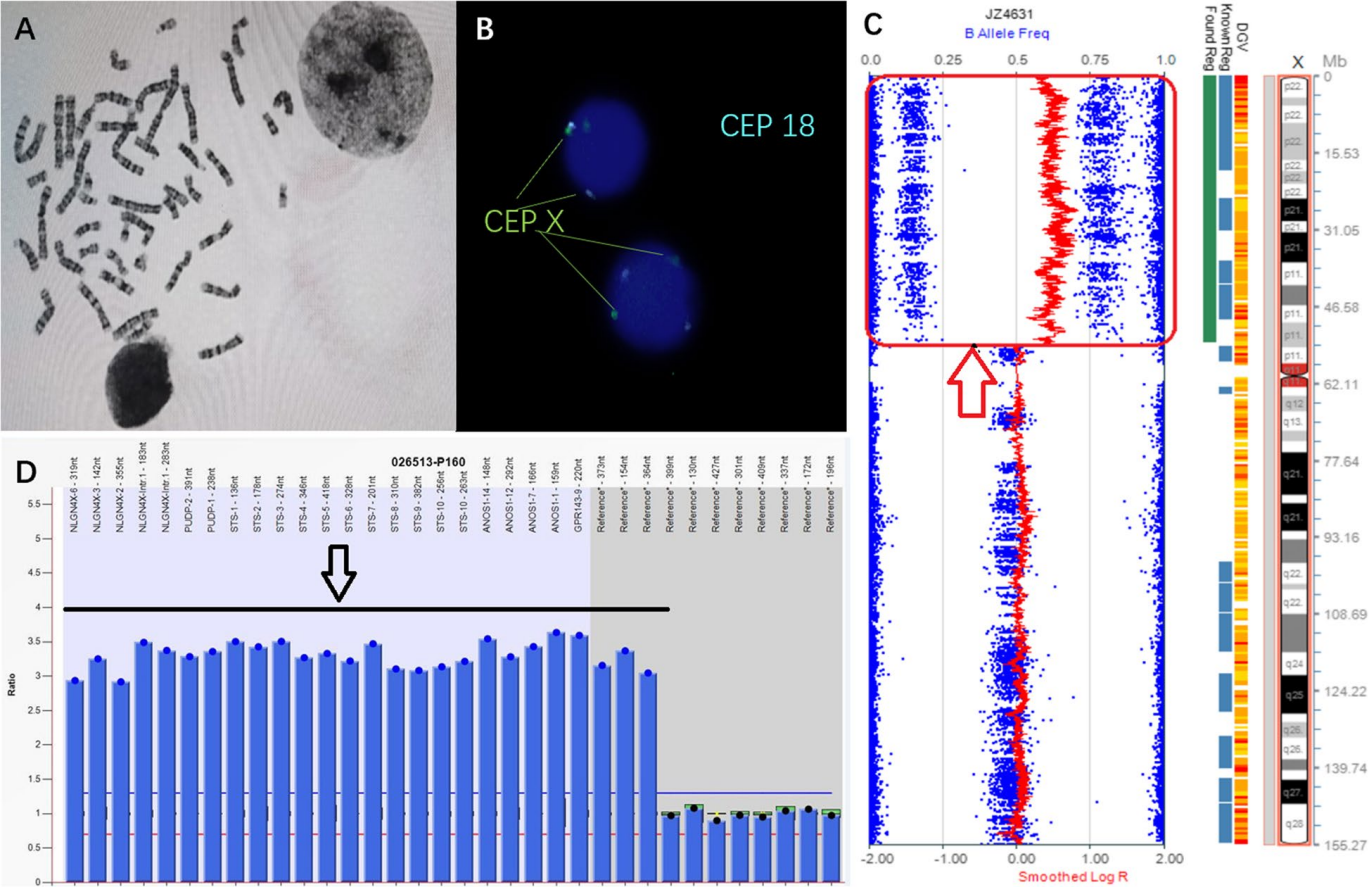
The detection of the foetus with the foetal cord blood sample. **A** Chromosomal G-banding of the foetus. **B** FISH results of interphase of cord blood cells of the foetus with the FIHS CEP X/Y18 probe. The green signals indicate CEP X, and the light blue signals indicate CEP 18. **C** The result of chromosomal microarray analysis (CMA) in the foetus. The CMA result revealed a recurrent CNV at Xp22.33-p11.22 (arr [hg19] Xp22.33-p11.22 (178,624-54,151,362) × 6), and the length of the duplication was 53.973 Mb (the relevant CNV region with a red arrow indicated). **D** The result of multiplex ligation-dependent probe amplification (MPLA) in the foetus. The MPLA result shows there are six duplications of CNVs at Xp22.33-p11.22 (the relevant CNV region with a black arrow indicated). The y-axis demonstrates the ratio signal compared to the normal control (ratio 1). The MLPA probes are displayed on the x-axis

This female foetus showed an approximate 8.5 mm primary atrial septal defect (Fig. 1A) and blood communication between the left and right atrium (Fig. 1B) at 21 weeks of gestation. A vascular shadow with an inner diameter of 2.6 mm was detected on the left side of the pulmonary artery (Fig. 1C), which merged into the dilated coronary sinus (Fig. 1D). A partial endocardial pad defect and a permanently dilated coronary sinus and left superior vena cava were diagnosed as foetal CHDs. Foetal cord blood samples were collected to measure the morphological structure of chromosomes, which depicted a normal karyotype (Fig. 2A). Furthermore, FISH depicted a normal result, indicating that the duplication region does not include the X centromere region (Fig. 2B). The peripheral blood of the parents was also used to analyse the karyotype, which were normal. The CMA results of the foetus demonstrated that there were recurrent CNVs at Xp22.33-p11.22 (arr [hg19] Xp22.33-p11.22 (178,624-54,151,362) × 6) (Fig. 2C). The pathological CNVs in this female foetus were approximately 53.973 Mb and encompassed 266 OMIM genes. It has been suggested that these CNVs are associated with CHD occurrence [2]. The duplications of recurrent CNVs were confirmed by MLPA. The results illustrate that there are six duplications in this region (Fig. 2D). To define the source of these recurrent pathological CNVs, the peripheral blood of the parents was collected for CMA analysis. The results showed that both parents had normal CMA results. In addition, based on guidelines for the interpretation of genetic variants [3] and similar CNV phenotypes observed in the literature [2], we classified the variant as clearly pathogenic based on the ACMG (https://www.acmg.net/)/ClinGen guidelines [4]. The couple was informed of the genetic test results and the meaning of the CNV findings, and in conjunction with the foetal ultrasound results and family history, they requested termination of the pregnancy based on informed consent.

## Discussion

In the present clinical case, the prenatal ultrasound results of the foetus illustrated a partial endocardial pad defect and a permanently dilated coronary sinus and left superior vena cava with, which were diagnosed as foetal CHDs (Fig. 1). We found pathological CNVs with six duplications at Xp22.33-p11.22 by CMA and MPLA, which is the first finding of CHDs with recurrent CNVs of six duplications. However, G-binding cytogenetic results demonstrated a normal karyotype in this female foetus. A previous study found that there was a pathogenic de novo CNV at Xp22.31 (6,455,151-8,132,677) in a foetus with a single ventricle, in which the size of the CNV was 1.68 Mb and the foetus had a normal karyotype [5]. We speculate that there might be a possible reason for this phenomenon, which is the centromere loss of the fragment during cell culture of samples. The centromere is a chromosomal structure that is critical for the accurate segregation of genetic information during mitosis and meiosis [6, 7]. It has been proven that the gain or loss of centromeres is important for genomic evolution and independent segregation of genes. In this case, the current fragment of CNVs on the X chromosome may have been lost due to the absence of centromeres when culturing foetal umbilical cord blood samples; as in conventional karyotyping procedures, blood samples undergo 2 growth cycles (72 hour) of in vitro amplification in the presence of culture media.

Chromosomal imbalance due to partial monosomy/trisomy, uniparental disomy (UPD), and activation of recessive gene pathogenic variants may cause clinical abnormalities through several different genetic mechanisms. Clinically significant and pathological CNVs were found in 21% of CHD patients [8]. A previous study reported mimic CNVs at Xp22.33-p11.22 in three foetuses from a family diagnosed with CHDs [2]. The first pregnancy was a dichorionic twin pregnancy with two male foetuses, and the second pregnancy had one male foetus. There is a rare novel 52.9 Mb CNVs of chromosome Xp22.33-p11.2 duplications in all three foetuses. They claimed that the foetus and parents had normal karyotypes. Their study revealed that a rare novel Xp22.33-p11.22 duplication might contribute to severe CHDs. The Xp22.33-p11.22 region contains several critical genes, such as steroid sulfatase (*STS*), anosmin 1 (*ANOS1*), X chromosomal neuroligins (*NLGN4X*), haloacid dehydrogenase-like hydrolase domain-containing protein 1 (*HDHD*1, also named *PUDP*) and G-protein coupled receptor 143 (*GPR143*) [9]. *STS* belongs to the sulfatase family and hydrolyses several 3β-hydroxysteroid sulphates, which serve as metabolic precursors for oestrogens, androgens, and cholesterol [10]. Large deletions/duplications of the *STS* gene are associated with X-linked ichthyosis (*XLI)* and are inherited in a recessive manner on the X chromosome [11]. Several previous studies have proven that the *STS* gene in the Xp22.3 chromosome is associated with some inherited diseases, including Turner syndrome and Klinefelter syndrome [12]. *ANOS1* is located on the X chromosome and can encode the extracellular glycoprotein anosmin-1. It has been shown that this protein plays an important role in central nervous system development, such as Kallmann syndrome (KS) [13]. More than 20 genes are related to KS. A previous study demonstrated that KS resulting from *ANOS1* pathogenic variant is an X-linked recessive inheritance disease [14]. Neuroligins (*NLGNs*) are postsynaptic cell adhesion molecules that have critical functions in synapse maturation, and *NLGNs* are located on the human X chromosome, which is called *NLGN4X* [15]. It has been demonstrated that variants in *NLGN4X* are a potential pathogenic mechanism for male bias in autism spectrum disorder [16, 17]. Otherwise, partial duplication of the *NLGN4X* gene (Xp22.32) is related to cognitive deficits [18]. *PUDP* encodes a member of the haloamide dehalogenase-like (*HAD*) hydrolase superfamily. Diseases associated with *PUDP* genes include ichthyosis, intellectual disability, X-linked and tricuspid valve stenosis [9, 19, 20]. Our MLPA results demonstrated six duplications in the exon region of *STS*, partial duplication in the exon and intron regions of *NLGN4X*, and the exon regions of *PUDP*, *ANOS1* and *GPR143* (Fig. 2D).

This study demonstrated that duplications at Xp22.33-p11.22 can be detected by CMA and MLPA but not by conventional cytogenetic methods with blood samples. Several cases have been reported where standard cytogenetic investigation failed to confirm true CNV in cell cultures established from foetal and placental tissues. In such a situation, CMA or MLPA analysis should provide a more effective means of assessing ploidy for specific chromosomes by offering the advantage of analysis of many nondividing cells. Furthermore, the recurrent and pathological CNVs of Xp22.33-p11.22 are associated with the occurrence of foetal CHDs. Future research into the functional influence of recurrent duplications will widen our knowledge and understanding of CHD aetiologies.

In conclusion, we reported and described six duplication CNVs in the Xp22.33-p11.22 region that were not detected by conventional cytogenetic methods. The novel finding will expand the understanding of the potential impact of this specific CNV on the patient’s health and confirm the reported pathogenic variants, which should help to improve genetic counselling, carrier identification and prenatal diagnosis in affected families and expand the insights of prenatal diagnosis of foetal CHDs.

## Data Availability

The datasets used and/or analysed during the current study are available from the corresponding author on reasonable request.

## Abbreviations

CNVs: Copy number variations
CMA: Chromosome Microarray Analysis
MLPA: Multiplex ligation-dependent probe amplification
CHDs: Congenital Heart Defects
SNPs: Single nucleotide polymorphisms

## References

[1] Eid OM, El Zomor H, Mohamed AM, El-Bassyouni HT, Afifi HH, El-Ayadi M, Sadek SH, Hammad SA, Salem SI, Mahrous R (2022). Multiplex ligation-dependent probe amplification versus fluorescent in situ hybridization for screening RB1 copy number variations in Egyptian patients with retinoblastoma. Ophthalmic Genet.

[2] Zhang J, Wu QQ, Wang L, Sun LJ (2015). A rare novel copy number variation of Xp22.33-p11.22 duplication is associated with congenital heart defects. Chin Med J.

[3] Richards S, Aziz N, Bale S, Bick D, Das S, Gastier-Foster J, Grody WW, Hegde M, Lyon E, Spector E (2015). Standards and guidelines for the interpretation of sequence variants: a joint consensus recommendation of the American College of Medical Genetics and Genomics and the Association for Molecular Pathology. Genetics Med.

[4] Riggs ER, Andersen EF, Cherry AM, Kantarci S, Kearney H, Patel A, Raca G, Ritter DI, South ST, Thorland EC (2020). Technical standards for the interpretation and reporting of constitutional copy-number variants: a joint consensus recommendation of the American College of Medical Genetics and Genomics (ACMG) and the clinical genome resource (ClinGen). Genet Med.

[5] Song T, Wan S, Li Y, Xu Y, Dang Y, Zheng Y, Li C, Zheng J, Chen B, Zhang J (2019). Detection of copy number variants using chromosomal microarray analysis for the prenatal diagnosis of congenital heart defects with normal karyotype. J Clin Lab Anal.

[6] Das A, Iwata-Otsubo A, Destouni A, Dawicki-McKenna JM, Boese KG, Black BE, Lampson MA (2022). Epigenetic, genetic and maternal effects enable stable centromere inheritance. Nat Cell Biol.

[7] Dong Q, Yang J, Gao J, Li F (2021). Recent insights into mechanisms preventing ectopic centromere formation. Open Biol.

[8] Nagy O, Szakszon K, Biro BO, Mogyorosy G, Nagy D, Nagy B, Balogh I, Ujfalusi A (2019). Copy number variants detection by microarray and multiplex ligation-dependent probe amplification in congenital heart diseases. J Biotechnol.

[9] Ma W, Mao J, Wang X, Duan L, Song Y, Lian X, Zheng J, Liu Z, Nie M, Wu X (2020). Novel microdeletion in the X chromosome leads to Kallmann syndrome, ichthyosis, obesity, and strabismus. Front Genet.

[10] Afzal S, Ramzan K, Ullah S, Wakil SM, Jamal A, Basit S, Waqar AB (2020). A novel nonsense mutation in the STS gene in a Pakistani family with X-linked recessive ichthyosis: including a very rare case of two homozygous female patients. BMC Med Genet.

[11] Boere PM, Bonnet C, Frausto RF, Fung SSM, Aldave AJ (2020). Multimodal imaging of pre-Descemet corneal dystrophy associated with X-linked ichthyosis and deletion of the STS gene. Cornea.

[12] Davies W (2021). The contribution of Xp22.31 gene dosage to turner and Klinefelter syndromes and sex-biased phenotypes. Eur J Med Genetics.

[13] Jiang X, Li D, Gao Y, Zhang X, Wang X, Yang Y, Shen Y (2020). A novel splice site variant in ANOS1 gene leads to Kallmann syndrome in three siblings. Gene.

[14] Topaloglu AK (2017). Update on the genetics of idiopathic hypogonadotropic hypogonadism. J Clin Res Pediatric Endocrinol.

[15] Zhang C, Milunsky JM, Newton S, Ko J, Zhao G, Maher TA, Tager-Flusberg H, Bolliger MF, Carter AS, Boucard AA (2009). A neuroligin-4 missense mutation associated with autism impairs neuroligin-4 folding and endoplasmic reticulum export. J Neurosci.

[16] Nguyen TA, Wu K, Pandey S, Lehr AW, Li Y, Bemben MA, Badger JD, Lauzon JL, Wang T, Zaghloul KA (2020). A cluster of autism-associated variants on X-linked NLGN4X functionally resemble NLGN4Y. Neuron.

[17] Kopp N, Amarillo I, Martinez-Agosto J, Quintero-Rivera F (2021). Pathogenic paternally inherited NLGN4X deletion in a female with autism spectrum disorder: clinical, cytogenetic, and molecular characterization. Am J Med Genet A.

[18] Magome T, Hattori T, Taniguchi M, Ishikawa T, Miyata S, Yamada K, Takamura H, Matsuzaki S, Ito A, Tohyama M (2013). XLMR protein related to neurite extension (Xpn/KIAA2022) regulates cell-cell and cell-matrix adhesion and migration. Neurochem Int.

[19] Todorova A, Litvinenko I, Todorov T, Tincheva R, Avdjieva D, Tincheva S, Mitev V (2014). A family with fragile X syndrome, Duchenne muscular dystrophy and ichthyosis transmitted by an asymptomatic carrier. Clin Genet.

[20] Labonne JDJ, Driessen TM, Harris ME, Kong IK, Brakta S, Theisen J, et al. Comparative Genomic Mapping Implicates LRRK2 for Intellectual Disability and Autism at 12q12, and HDHD1, as Well as PNPLA4, for X-Linked Intellectual Disability at Xp22.31. J Clin Med. 2020;9(1):274. 10.3390/jcm9010274 PMC7019335 31963867

